# Association of SGLT2 Inhibitors with Mortality and Bioprosthesis Valve Failure After TAVR: A Propensity-Matched Cohort Study

**DOI:** 10.3390/jcm14197001

**Published:** 2025-10-03

**Authors:** Olivier Morel, Amandine Granier, Lisa Lochon, Antonin Trimaille, Arnaud Bisson, Benjamin Marchandot, Anne Bernard, Laurent Fauchier

**Affiliations:** 1 Department of Cardiovascular Medicine, Nouvel Hôpital Civil, Strasbourg University Hospital, 67098 Strasbourg, France; amandine.granier@chru-strasbourg.fr (A.G.); antonin.trimaille@gmail.com (A.T.);; 2 UR 3074 Translational Cardiovascular Medicine, CRBS, University of Strasbourg, 67000 Strasbourg, France; 3 Hanoï Medical University, Hanoi 10000, Vietnam; 4 Service de Cardiologie, Centre Hospitalier Universitaire Trousseau et Faculté de Médecine, Université François Rabelais, 37000 Tours, France; lisa.lochon@chu-tours.fr (L.L.); a.bisson@chu-tours.fr (A.B.); anne.bernard@univ-tours.fr (A.B.); laurent.fauchier@univ-tours.fr (L.F.); 5 Inserm U1327 ISCHEMIA “Membrane Signalling and Inflammation in Reperfusion Injuries”, Université de Tours, 37000 Tours, France

**Keywords:** TAVR, bioprosthesis dysfunction, valve, aortic stenosis, TAVI

## Abstract

**Highlights:**

**What is the clinical question being addressed?**

- Can SGLT2 inhibitors improve clinical outcomes in patients undergoing transcatheter aortic valve replacement (TAVR), including reducing the risk of bioprosthetic valve failure?

**Key finding?**

- In patients undergoing TAVR, the use of SGLT2 inhibitors was associated with lower risks of mortality and bioprosthetic valve failure. These findings suggest a potential disease-modifying role for SGLT2 inhibitors in this population.

**Abstract:**

**Background:** Sodium–glucose cotransporter 2 inhibitors (SGLT2i) have shown cardioprotective effects beyond glucose control. In aortic stenosis, SGLT2 expression is upregulated in myocardium and valve tissue, contributing to inflammation, oxidative stress, thrombogenicity, and calcification. SGLT2 inhibition may counteract these mechanisms, potentially reducing bioprosthetic valve failure after transcatheter aortic valve replacement (TAVR), where the diseased native valve remains in place. **Objectives:** This study aimed to evaluate whether SGLT2i use is associated with improved clinical outcomes, including all-cause mortality and bioprosthetic valve failure, following TAVR. **Methods:** We conducted a retrospective cohort study using the TriNetX global health research network. Adults with non-rheumatic aortic stenosis who underwent TAVR were stratified by SGLT2i use. Propensity score matching (1:1) was applied to balance baseline characteristics (n = 2297 per group). Primary outcomes were all-cause mortality and bioprosthetic valve failure during follow-up. **Results:** Before matching, SGLT2i users had more cardiovascular comorbidities. After matching, SGLT2i use was associated with a significantly lower risk of all-cause mortality (HR: 0.83; 95% CI: 0.71–0.97; *p* = 0.02) and bioprosthetic valve failure (HR: 0.62; 95% CI: 0.39–0.99; *p* = 0.04). **Conclusions:** In a large real-world cohort of TAVR recipients, SGLT2i use was independently associated with reduced mortality and lower risk of bioprosthetic valve failure. These findings support a potential disease-modifying role for SGLT2 inhibitors in this high-risk population and warrant further prospective investigation.

## 1. Introduction

Sodium–glucose cotransporter 2 inhibitors (SGLT2i), initially developed for the treatment of type 2 diabetes mellitus, have demonstrated significant cardiovascular benefits, particularly in patients with heart failure [1]. Beyond their glucose-lowering properties, SGLT2i exert pleiotropic actions that may be relevant to valvular heart disease [2,3]. In patients with aortic stenosis (AS), upregulation of the SGLT2 gene and protein has been observed in cardiomyocytes and is associated with myocardial fibrosis, inflammation, and oxidative stress [4]. Furthermore, recent evidence indicates that SGLT2 is expressed in calcified human aortic valves, colocalizing with inflammatory and osteogenic activity [5]. Mechanistic studies suggest that SGLT2 inhibition may attenuate oxidative stress, improve endothelial function, suppress proinflammatory signaling, and reduce fibrotic remodeling—key mechanisms implicated in bioprosthetic valve failure [5]. These effects may be particularly relevant in the context of transcatheter aortic valve replacement (TAVR), where the native valve remains in situ, potentially interacting with the implanted prosthesis [6,7,8]. Given these mechanistic insights, we sought to determine whether SGLT2i use is associated with improved clinical outcomes after TAVR, with a focus on all-cause mortality and bioprosthetic valve failure.

## 2. Methods

We analyzed data from the TriNetX research network to identify TAVR patients, comparing outcomes between those treated with SGLT2i and non-users. TriNetX is a global federated health research network that provides access to electronic medical records (EMRs) from participating 147 health care organizations in 18 countries, covering approximately 150 million individuals. In this retrospective observational study, available data included demographics, diagnoses based on the International Classification of Diseases (ICD-10-CM) codes, and medications classified using the Anatomical Therapeutic Chemical Classification (ATC) System. This research network has been previously used to study patients with diabetes and cardiovascular conditions [9].

All statistical analyses were performed on the TriNetX platform. Inclusion criteria consisted of TAVR (ICD-10-CM: 135.0). Mechanical complications of bioprosthetic valves were identified using the 2025 ICD-10 diagnosis code T85.6. While this coding does not capture the full spectrum of hemodynamic structural valve dysfunction, it reflects clinically meaningful events in line with the EAPCI classification, which stages bioprosthetic valve failure (BVF) as follows:

Stage 1: BVF with clinical symptoms or irreversible stage 3 severe hemodynamic deterioration;

Stage 2: BVF requiring aortic valve reintervention;

Stage 3: BVF resulting in aortic valve-related death [8].

Exclusion criteria included a history of endocarditis, aortocoronary bypass graft surgery, and the presence of prosthetic or xenogeneic heart valves. Balanced cohorts were created using 1:1 propensity score matching (PSM) with greedy nearest neighbor matching. Absolute standardized mean differences (SDs) were used to show the distribution of demographic and clinical data among the groups and were calculated as the differences in the means or proportions of a particular variable divided by the pooled estimate of SD for that variable. Any baseline characteristic with an SD of < 10% was considered well-matched. Covariates for matching included demographics, cardiovascular and non-cardiovascular comorbidities, use of medications, and several values of laboratory tests (Table 1). Patients were stratified by SGLT2i use, and 1:1 propensity score matching was applied to balance baseline characteristics (n = 2297 per group). After PSM, Cox proportional hazard models were used to calculate hazard ratios (HRs) and 95% confidence intervals (95% CI) for the risk of adverse events in patients treated with SGLT2 inhibitors.

This research complies with data governance policies: studies using TriNetX do not require institutional ethical approval as no identifiable patient data are accessed, and publication agreements are in place with contributing healthcare organizations. The primary outcomes were all-cause mortality and bioprosthetic valve failure during follow-up [8].

## 3. Results

Outcomes were assessed over 1.4 ± 1 years, with a median 1.2 IQR 1.8 follow-up period. Among 26,944 patients who underwent TAVR, 2372 (8.8%) received SGLT2 inhibitor therapy. Prior to PSM, SGLT2i users were younger and had a higher prevalence of cardiovascular risk factors and comorbidities, including diabetes mellitus, heart failure, coronary artery disease, peripheral artery disease, atrial fibrillation, and chronic kidney disease (Table 1). Baseline clinical characteristics before and after PSM are also presented in Table 1. Most patients were treated with beta-blockers (83%), calcium channel blockers (73%), ACE inhibitors (54%), angiotensin II receptor blockers (52%), diuretics (88%), and mineralocorticoid receptor antagonists (39%).

**Figure 1:**
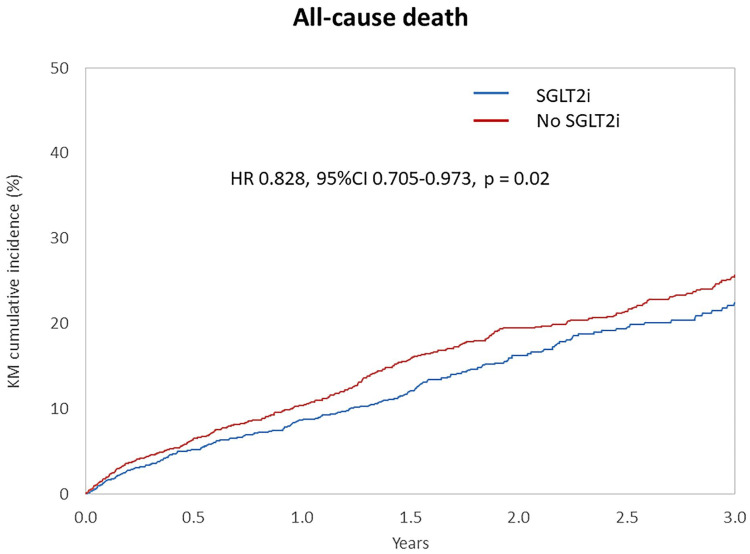
Cumulative incidence of all-cause mortality in patients treated or not by SGLT2 inhibitors after TAVR.

**Figure 2:**
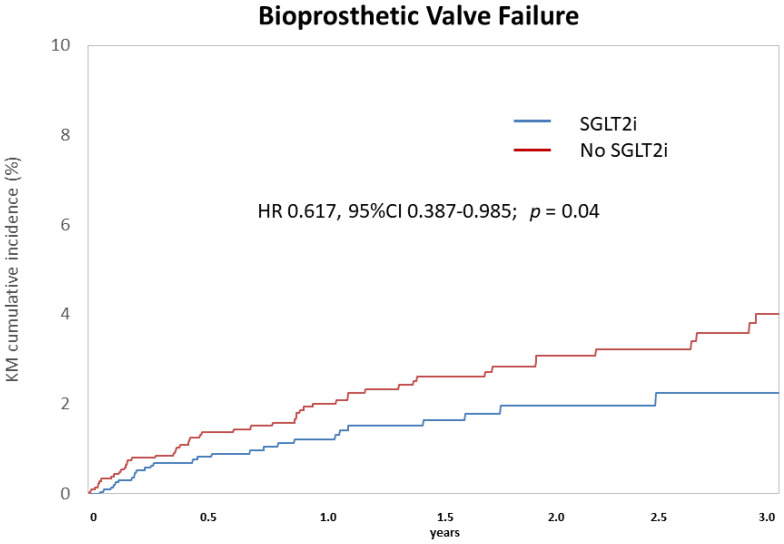
Cumulative incidence of bioprosthetic valve failure in patients treated or not by SGLT2 inhibitors after TAVR.

Following matching, the annual mortality rate was 7.51% among SGLT2i users (n = 2297) compared to 8.57% in non-users (n = 2297). The annual rate of bioprosthetic valve failure was 0.75% in the SGLT2i group versus 1.34% in controls. SGLT2i use was associated with a significantly lower risk of all-cause mortality (hazard ratio [HR]: 0.83; 95% confidence interval [CI]: 0.71–0.97; *p* = 0.02) (Figure 1) and bioprosthetic valve failure (HR: 0.62; 95% CI: 0.39–0.99; *p* = 0.04) (Figure 2).

Incident atrial fibrillation was numerically lower in SGLT2i users but did not reach statistical significance (6.4% vs. 7.5%, *p* = 0.06). In contrast, no significant differences were observed between groups in the incidence of thrombotic events, major bleeding, renal dysfunction, endocarditis, or heart failure (Table 2).

## 4. Discussion

In this large, real-world cohort study of patients undergoing TAVR, use of SGLT2 inhibitors was independently associated with reduced all-cause mortality and lower rates of bioprosthetic valve failure, even after rigorous adjustment for baseline comorbidities. These findings suggest a potential cardioprotective and valve-modifying role for SGLT2i in this high-risk population.

Recent insights have led to a paradigm shift in the understanding of aortic stenosis, now recognized as a disease involving both the valve and the ventricle, rather than an isolated pathology of the aortic valve apparatus [10]. While the expression of SGLT2 in the cardiovascular system was previously uncertain, emerging evidence has firmly established its presence and identified subclinical inflammation as a key driver of SGLT2 overexpression [11,12]. In the context of AS, we have recently demonstrated that plasma from patients with severe AS exhibits a distinct proinflammatory phenotype, characterized by elevated levels of cytokines and factor Xa activity [13]. Compared with plasma from healthy individuals or patients with cardiovascular risk factors but no AS, plasma from AS patients markedly increases oxidative stress in valvular endothelial cells (VECs). This oxidative burden leads to endothelial dysfunction via SGLT2 overexpression in VECs, promoting a prothrombotic, proinflammatory, and proadhesive phenotype—an effect that is attenuated by empagliflozin, a selective SGLT2 inhibitor [13]. SGLT2 expression is also upregulated in the myocardium, particularly in patients with low-flow, low-gradient (LF/LG) AS, where it correlates with oxidative stress, myocardial fibrosis, and inflammation. In calcified human aortic valves, SGLT2 colocalizes with markers of oxidative stress and inflammation, further implicating its role in disease progression [4]. Moreover, extracellular vesicles (EVs) derived from platelets, leukocytes, and endothelial cells—found in high concentrations in AS plasma and within stenotic valves—have been identified as potent inducers of SGLT2 expression [5,14,15,16,17,18]. These EVs are associated with enhanced thrombogenicity and have been proposed as biomarkers of adverse outcomes following TAVR [19], underscoring their potential role in valvular degeneration and post-TAVR complications [5,6,8].

Recent data from small cohort studies have convincingly demonstrated the favorable impact of SGLT2 inhibitor use following TAVR in diabetic patients, showing improved cardiac remodeling and a reduced risk of major adverse cardiovascular events (MACEs), including all-cause mortality (HR: 0.51; 95% CI: 0.25–0.98; *p* < 0.001) and heart failure hospitalization (HR: 0.40; 95% CI: 0.07–0.62; *p* = 0.009) at 2-year follow-up [20]. Moreover, a recent randomized trial of 1222 older adults with aortic stenosis undergoing TAVR and at high risk for heart failure events demonstrated that dapagliflozin significantly reduced the risk of heart failure worsening (HR: 0.63; 95% CI: 0.45–0.88), although it did not result in a statistically significant reduction in all-cause mortality (HR: 0.87; 95% CI: 0.59–1.28) [21]. In line with these findings, our nationwide registry-based analysis identified a significant reduction in all-cause mortality among SGLT2i users, further supporting the safety and potential efficacy of SGLT2 inhibitors in the TAVR population. The absence of a significant impact on heart failure outcomes in our study may reflect residual confounding, particularly the higher prevalence of heart failure with preserved ejection fraction (HFpEF) or a greater proportion of low-flow, low-gradient (LFLG) aortic stenosis among SGLT2i users—both of which are frequent clinical indications for initiating SGLT2i therapy.

Beyond their effects on myocardial outcomes, recent analyses from small observational cohorts have suggested that SGLT2 inhibitor use may slow the progression of aortic stenosis in patients with moderate disease [22]. Indeed, a recent post hoc analysis of a cohort comprising 458 patients treated with SGLT2 inhibitors and 11,240 who were never prescribed these agents found that, after adjusting for time-varying exposure, relevant covariates, and competing risks, patients receiving SGLT2 inhibitors had a significantly lower likelihood of progressing from non-severe to severe aortic stenosis (HR: 0.61; 95% CI: 0.39–0.94; *p* = 0.03) over a median follow-up of 3.4 years. Further, it appeared that the longer the treatment, the slower the progression (HRs: 0.54, 0.48, and 0.27, for 3-, 6-, and 12-month treatment duration) [22].

Aligned with this emerging paradigm, our study provides novel evidence that SGLT2 inhibitor use may also be associated with a reduced risk of bioprosthetic valve failure following TAVR. This finding is biologically plausible, supported by mechanistic data indicating that the native valve remains biologically active, as emphasized by 18F-sodium fluoride positron emission tomography (18F-NaF PET) uptake, a marker of calcification activity and vascular injury, and may contribute to bioprosthetic valve deterioration [8,23]. SGLT2 is expressed within calcified human aortic valves, where it colocalizes with markers of inflammation, oxidative stress, and osteogenic activity [5]. Experimental studies have shown that SGLT2 inhibition can attenuate valvular calcification by suppressing proinflammatory signaling, oxidative stress, and fibrotic remodeling—key processes implicated in structural valve degeneration [5]. Important insights into the determinants of structural valve dysfunction have been gained from systematic ex vivo analyses of explanted transcatheter heart valves [24]. A previous report demonstrated a time-dependent pattern of THV degeneration, beginning with thrombus formation within hours, followed by endothelial hyperplasia, inflammation, and fibrosis by 60 days, and ultimately progressing to tissue remodeling and calcification after four years [24]. Recent analyses by Sato and colleagues have highlighted that leaflet thickening—a hallmark of valve dysfunction—corresponds histologically to acute, organizing, and organized thrombus (i.e., pannus formation) [25]. According to this perspective, early valve thrombosis likely constitutes an active nidus that initiates and promotes subsequent valve remodeling, including leaflet thickening and the development of calcification [8,26]. Notably, these pathological mechanisms, including platelet adhesion and thrombin generation, may be attenuated by SGLT2 inhibition [5,12]. Given the relatively short follow-up duration in this study (median 1.2 years), we cannot exclude the possibility that the primary valvulo-protective effects of SGLT2i are driven by the inhibition of thrombotic processes. Other potential benefits of valve remodeling, such as reductions in fibrosis and calcification, are likely to require a longer time to manifest [24]. In the specific context of TAVR, where the native valve remains in place and continues to interact with the implanted bioprosthesis, the protective mechanisms conferred by SGLT2 inhibition may be particularly relevant [27]. Our findings suggest that SGLT2 inhibitors may exert not only cardioprotective but also valve-protective effects, potentially enhancing bioprosthesis durability in selected patients. Supporting this hypothesis, a recent retrospective analysis of 1838 adults who underwent aortic valve replacement—either TAVR or surgical (SAVR)—demonstrated a significantly lower incidence of bioprosthetic valve dysfunction among SGLT2 inhibitor users. Over a median follow-up of 4.85 years, SGLT2 inhibitor use was associated with a markedly reduced risk of valve dysfunction (HR: 0.37; 95% CI: 0.18–0.78; *p* = 0.008) [25]. Whether this protective effect is more pronounced in TAVR—where the SGLT2-rich native valve is preserved—compared to SAVR, where the valve is excised, remains to be clarified. Collectively, these data support the hypothesis that the progression of both native and bioprosthetic valve degeneration may be modifiable through SGLT2 inhibition [3,22,28]. Future multimodal studies combining circulating biomarkers with advanced imaging, such as 18F-sodium fluoride PET, could help validate whether the pleiotropic effects of SGLT2i mitigate native valve–bioprosthesis interactions and reduce valve dysfunction [23,26]

### Study Limitations

This study has several important limitations. First, its retrospective observational design precludes definitive conclusions about causality, and residual confounding cannot be excluded despite rigorous propensity score matching. Second, medication exposure was based on prescription records, which may not fully reflect actual patient adherence or treatment duration. While such data may not fully capture individual adherence, prior validation studies support the use of prescription and refill records as a reliable proxy for medication exposure in large cohorts such as the one analyzed in this study. Regarding dosing, SGLT2 inhibitors are typically prescribed at a fixed dose of 10 mg daily, with no routine titration; therefore, dose variability did not affect our analysis. It is also worth noting that any potential misadherence to SGLT2i would most likely lead to an underestimation of the observed protective effects on overall mortality and valve failure. Third, detailed hemodynamic and echocardiographic data, including valve gradients, effective orifice area, and markers of prosthetic valve hemodynamics, were not consistently available across the dataset, limiting assessment of subclinical bioprosthetic dysfunction. Importantly, hemodynamic structural valve dysfunction without significant clinical manifestations could not be fully captured in this investigation. Fourth, the definition of bioprosthetic valve failure was based on diagnostic coding, which may lack granularity and be subject to misclassification. Fifth, key procedural details—such as valve type (self-expanding vs. balloon-expandable, intra-annular vs. supra-annular), as well as other factors known to influence valve durability, including valve asymmetry and commissural alignment—were not recorded in the TriNetX database. This limitation is particularly important when investigating the mechanisms underlying valve durability. Sixth, the findings are derived from a real-world registry encompassing heterogeneous clinical practices, which may affect generalizability. Although TriNetX provides access to a large, multinational cohort, selection bias from predominantly high-income countries may limit generalizability. Replication in underrepresented regions is warranted. Lastly, as the study relied on routinely collected electronic health record data, certain variables—such as imaging biomarkers (e.g., 18F-NaF PET), valve type, procedural details, and laboratory parameters—were unavailable, limiting mechanistic insight. Prospective, randomized studies are needed to confirm these associations and elucidate underlying mechanisms.

## 5. Conclusions

In a large real-world cohort of TAVR recipients, SGLT2i use was associated with reduced risks of mortality and bioprosthetic valve failure. These findings suggest a potential disease-modifying effect of SGLT2 inhibitors in this population and warrant further investigation in prospective studies.

## Figures and Tables

**Table 1:** Baseline characteristics of patients with TAVR before and after propensity score matching.

	Before Propensity Score Matching			After Propensity Score Matching		
	SGLT2i Post TAVR	No SGLT2i Post TAVR	Std Diff. (%)	SGLT2i Post TAVR	No SGLT2i Post TAVR	Std Diff. (%)
	(n = 2372)	(n = 24,572)		(n = 2297)	(n = 2297)	
Demographics						
Age (years), mean ± SD	75.9 ± 8.9	78.0 ± 8.5	23.9	76.0 ± 8.8	76.1 ± 9.3	0.3
Men, n (%)	1441 (60.8%)	13,136 (53.5%)	14.8	1385 (60.3%)	1381 (60.1%)	0.4
White, n (%)	1899 (80.1%)	20,382 (82.9%)	7.4	1843 (80.2%)	1863 (81.1%)	2.2
Black or African American, n (%)	166 (7%)	1146 (4.7%)	10	160 (7%)	152 (6.6%)	1.4
Asian, n (%)	90 (3.8%)	695 (2.8%)	5.4	87 (3.8%)	72 (3.1%)	3.6
Hispanic or Latino, n (%)	116 (4.9%)	770 (3.1%)	9	107 (4.7%)	114 (5%)	1.4
Risk factors						
Hypertension, n (%)	2036 (85.8%)	20,961 (85.3%)	1.5	1976 (86%)	1973 (85.9%)	0.4
Diabetes mellitus, n (%)	1675 (70.6%)	9833 (40%)	64.7	1611 (70.1%)	1667 (72.6%)	5.4
Smoker, n (%)	1079 (45.5%)	9555 (38.9%)	13.4	1047 (45.6%)	1034 (45%)	1.1
Overweight or obesity, n (%)	1148 (48.4%)	9164 (37.3%)	22.6	1113 (48.5%)	1126 (49%)	1.1
Dyslipidemia, n (%)	2071 (87.3%)	20,710 (84.3%)	8.7	2006 (87.3%)	1998 (87%)	1
Alcohol related diagnoses, n (%)	83 (3.5%)	617 (2.5%)	5.8	81 (3.5%)	85 (3.7%)	0.9
Cardiovascular comorbidities						
Heart failure, n (%)	2147 (90.5%)	18,061 (73.5%)	45.4	2072 (90.2%)	2092 (91.1%)	3
Coronary artery disease, n (%)	2130 (89.8%)	20,698 (84.2%)	16.6	2059 (89.6%)	2062 (89.8%)	0.4
Myocardial infarction, n (%)	680 (28.7%)	4117 (16.8%)	28.7	653 (28.4%)	636 (27.7%)	1.6
Ischemic stroke, n (%)	325 (13.7%)	2499 (10.2%)	10.9	311 (13.5%)	322 (14%)	1.4
Intracranial hemorrhage, n (%)	18 (0.8%)	194 (0.8%)	0.3	17 (0.7%)	20 (0.9%)	1.5
Atrial fibrillation or flutter, n (%)	1303 (54.9%)	10,481 (42.7%)	24.8	1257 (54.7%)	1246 (54.2%)	1
Peripheral vascular disease, n (%)	521 (22%)	4459 (18.1%)	9.5	501 (21.8%)	493 (21.5%)	0.8
Non-cardiovascular comorbidities						
Kidney disease, n (%)	1487 (62.7%)	10,765 (43.8%)	38.5	1433 (62.4%)	1475 (64.2%)	3.8
COPD, n (%)	613 (25.8%)	4919 (20%)	13.9	596 (25.9%)	565 (24.6%)	3.1
Sleep apnea syndrome, n (%)	876 (36.9%)	6125 (24.9%)	26.2	840 (36.6%)	872 (38%)	2.9
Previous cancer, n (%)	926 (39%)	9112 (37.1%)	4	899 (39.1%)	930 (40.5%)	2.8
Cognitive impairment, n (%)	26 (1.1%)	280 (1.1%)	0.4	25 (1.1%)	27 (1.2%)	0.8
Laboratory tests and examinations						
Body mass index (kg/m2), mean ± SD	30.2 ± 7.1	29.3 ± 6.6	13.2	30.2 ± 7.1	30.3 ± 6.8	1.3
Total cholesterol (mg/dL), mean ± SD	139.8 ± 39.0	150.5 ± 41.3	26.6	140.0 ± 39.0	139.5 ± 37.2	1.4
LDL cholesterol (mg/dL), mean ± SD	70.8 ± 30.6	78.3 ± 32.7	23.6	70.9 ± 30.6	71.2 ± 29.8	0.8
HDL cholesterol (mg/dL), mean ± SD	42.0 ± 17.5	45.4 ± 20.6	17.9	42.1 ± 17.6	41.8 ± 18.1	1.6
Triglyceride (mg/dL), mean ± SD	129.6 ± 95.4	119.2 ± 68.6	12.5	129.8 ± 95.8	125.9 ± 75.1	4.5
HbA1c >= 6%, n (%)	1368 (57.7%)	7982 (32.5%)	52.3	1323 (57.6%)	1364 (59.4%)	3.6
Estimated GFR (MDRD, mL/min), mean ± SD	61.0 ± 25.4	65.1 ± 26.2	15.7	61.0 ± 25.1	59.9 ± 26.9	3.9
BNP, ng/L, mean ± SD	1056.9 ± 2389.7	1109.8 ± 4254.2	1.5	1069.6 ± 2427.7	927.0 ± 1902.4	6.5
NT-proBNP, ng/L, mean ± SD	4487.0 ± 7191.7	3647.5 ± 8391.8	10.7	4480.4 ± 7215.8	4984.5 ± 9637.5	5.9
LVEF, mean ± SD	50.9 ± 16.1	58.7 ± 11.4	56	51.3 ± 16.0	51.9 ± 15.4	3.8
Medications						
Beta Blockers, n (%)	1989 (83.9%)	17,417 (70.9%)	31.4	1916 (83.4%)	1929 (84%)	1.5
Calcium Channel Blockers, n (%)	1742 (73.4%)	17,963 (73.1%)	0.8	1688 (73.5%)	1682 (73.2%)	0.6
ACE Inhibitors, n (%)	894 (37.7%)	7865 (32%)	11.9	858 (37.4%)	893 (38.9%)	3.1
Angiotensin II receptor blockers, n (%)	1325 (55.9%)	8580 (34.9%)	43	1257 (54.7%)	1258 (54.8%)	0.1
MRA, n (%)	969 (40.9%)	3074 (12.5%)	67.6	901 (39.2%)	870 (37.9%)	2.8
Digitalis glycosides, n (%)	187 (7.9%)	1076 (4.4%)	14.6	177 (7.7%)	205 (8.9%)	4.4
Diuretics, n (%)	2115 (89.2%)	17,019 (69.3%)	50.6	2040 (88.8%)	2088 (90.9%)	6.9
Lipid-lowering drugs, n (%)	2199 (92.7%)	20,741 (84.4%)	26.3	2127 (92.6%)	2140 (93.2%)	2.2
Insulin, n (%)	696 (29.3%)	3195 (13%)	40.8	666 (29%)	696 (30.3%)	2.9
Metformin, n (%)	869 (36.6%)	3578 (14.6%)	52.3	824 (35.9%)	850 (37%)	2.4
Sulfonylureas, n (%)	460 (19.4%)	1718 (7%)	37.3	436 (19%)	473 (20.6%)	4
GLP-1 receptor agonists, n (%)	365 (15.4%)	926 (3.8%)	40.3	336 (14.6%)	333 (14.5%)	0.4
DPP4 inhibitors, n (%)	277 (11.7%)	925 (3.8%)	30	261 (11.4%)	258 (11.2%)	0.4
Thiazolidinediones, n (%)	105 (4.4%)	289 (1.2%)	19.8	96 (4.2%)	98 (4.3%)	0.4
Antiplatelet therapy, n (%)	2320 (97.8%)	23,968 (97.5%)	1.8	2246 (97.8%)	2248 (97.9%)	0.6
Anticoagulant, n (%)	1052 (44.4%)	7718 (31.4%)	26.9	1012 (44.1%)	1019 (44.4%)	0.6

Values are n (%) or mean ± SD. ACE, angiotensin converting enzyme; COPD, chronic obstructive pulmonary disease; DPP4, dipeptidyl peptidase-4; LVEF, left ventricular ejection fraction; SD, standard deviation; SGLT2i, sodium–glucose cotransporter 2 inhibitor.

**Table 2:** Clinical outcomes during FU in the matched population of patients with TAVR (FU 1.4 ± 1 years, median 1.2, IQR 1.8).

	SGLT2i Post TAVR		No SGLT2i Post TAVR		Hazard Ratio (95% CI)	*p* Value
	(n = 2297)		(n = 2297)			
	Number of Events	Yearly Rate, %	Number of Events	Yearly Rate, %		
Death	250	7.51	370	8.57	0.828 (0.705–0.973)	0.02
Bioprosthetic valve failure	27	0.75	51	1.34	0.617 (0.387–0.985)	0.04
Incident cardiogenic shock	50	1.70	47	1.49	1.338 (0.897–1.997)	0.15
Incident acute pulmonary edema	43	1.42	39	1.10	1.323 (0.857–2.045)	0.21
Incident HF	26	6.10	35	10.58	0.76 (0.457–1.263)	0.29
Ischemic stroke or thromboembolism	95	4.01	105	3.56	1.113 (0.842–1.471)	0.45
Major bleeding	444	11.70	550	12.27	0.925 (0.816–1.049)	0.22
Acute MI	62	1.63	81	1.99	0.893 (0.641–1.245)	0.51
Pacemaker or ICD implantation	97	3.70	97	3.45	1.204 (0.908–1.597)	0.2
AV block	80	4.11	85	3.56	1.153 (0.848–1.566)	0.36
Incident AF	90	6.46	140	7.53	0.776 (0.595–1.011)	0.06
VT/VF/Cardiac arrest	252	6.47	274	5.88	1.054 (0.888–1.251)	0.55
MI/stroke/HF/death	815	15.68	868	16.45	1.047 (0.952–1.153)	0.35
ESKD	29	1.08	34	1.16	1.087 (0.660–1.788)	0.74
Incident cancer	118	7.83	138	7.40	1.014 (0.792–1.297)	0.91
Incident endocarditis	58	2.30	75	2.16	0.933 (0.661–1.316)	0.69

AF, atrial fibrillation; ESKD, end-stage kidney disease; HF, heart failure; MI, myocardial infarction; TAVR, transcatheter aortic valve replacement; VF, ventricular fibrillation; VT, ventricular tachycardia.

## Data Availability

The original contributions presented in this study are included in the article. Further inquiries can be directed to the corresponding author.
